# Evaluation of chronic statin treatment on sepsis outcome in I.C.U

**DOI:** 10.1186/2197-425X-3-S1-A872

**Published:** 2015-10-01

**Authors:** HAA Youssef, HM Koptan, A Moustafa

**Affiliations:** Faculty of Medicine, Menoufyia University, Anesthesia, Menoufia, Egypt

Severe sepsis and septic shock are common and frequently fatal problem in I.C.U. Recent therapeutic advances to routine clinical practice has proven controversial, because of their pleiotropic effects related to many pathophysiological determinants of sepsis. Statin therapy could be the next step in the search for adjuvant therapy. This study was done to evaluate chronic statin therapy on outcome of patients with sepsis and septic shock in I.C.U

## Methods

We compared patients with severe sepsis and septic shock. Two groups were identified, group A, patients didn`t receive statin before or during their I.C.U management. Group B, patients with ongoing statin therapy before admission and continued during I.C.U therapy. The primary end point was the number of organ failure free days, hemodynamic failure free days and organ dysfunction free days up to day 14. Secondary endpoints included hospital mortality and safety.

## Results

Patients in whom chronic statin therapy had been continued in the I.C.U (n = 45) had significantly more organ failure free days 12(5-15), organ dysfunction free days 10(2-13), hemodynamic failure free days 13(9-14) as compared to others non statin group(n = 33) with organ failure fee days 5(0-11), organ dysfunction free days 2(0-10), and hemodynamic failure free days 9(6-12). The need for increased mortality and hospital length of stay were comparable in the two groups.

## Conclusions

Chronic statin therapy in I.C.U septic patients was associated with reduction in the severity of organ failure and hemodynamic failure with no improvement in hospital mortality and length of stay.
